# FGF21 ameliorates the neurocontrol of blood pressure in the high fructose-drinking rats

**DOI:** 10.1038/srep29582

**Published:** 2016-07-08

**Authors:** Jian-Li He, Miao Zhao, Jing-Jun Xia, Jian Guan, Yang Liu, Lu-Qi Wang, Dong-Xue Song, Mei-Yu Qu, Meng Zuo, Xin Wen, Xue Yu, Rong Huo, Zhen-Wei Pan, Tao Ban, Yan Zhang, Jiu-Xin Zhu, Weinian Shou, Guo-Fen Qiao, Bai-Yan Li

**Affiliations:** 1 Department of Pharmacology (State-Province Key Laboratories of Biomedicine-Pharmaceutics of China, Key Laboratory of Cardiovascular Medicine Research, Ministry of Education), College of Pharmacy, Harbin Medical University, Harbin, China; 2 Department of Orthopedics, the Second Affiliated Hospital of Harbin Medical University, Harbin, China; 3 Riley Heart Research Center, Division of Pediatric Cardiology, Herman B. Wells Center for Pediatric Research, Department of Pediatrics, Indiana University School of Medicine, Indianapolis, USA

## Abstract

Fibroblast growth factor-21 (FGF21) is closely related to various metabolic and cardiovascular disorders. However, the direct targets and mechanisms linking FGF21 to blood pressure control and hypertension are still elusive. Here we demonstrated a novel regulatory function of FGF21 in the baroreflex afferent pathway (the nucleus tractus solitarii, NTS; nodose ganglion, NG). As the critical co-receptor of FGF21, β-klotho (klb) significantly expressed on the NTS and NG. Furthermore, we evaluated the beneficial effects of chronic intraperitoneal infusion of recombinant human FGF21 (rhFGF21) on the dysregulated systolic blood pressure, cardiac parameters, baroreflex sensitivity (BRS) and hyperinsulinemia in the high fructose-drinking (HFD) rats. The BRS up-regulation is associated with Akt-eNOS-NO signaling activation in the NTS and NG induced by acute intravenous rhFGF21 administration in HFD and control rats. Moreover, the expressions of FGF21 receptors were aberrantly down-regulated in HFD rats. In addition, the up-regulated peroxisome proliferator-activated receptor-γ and -α (PPAR-γ/-α) in the NTS and NG in HFD rats were markedly reversed by chronic rhFGF21 infusion. Our study extends the work of the FGF21 actions on the neurocontrol of blood pressure regulations through baroreflex afferent pathway in HFD rats.

The fibroblast growth factor-21 (FGF21) is a master regulator for its effects on metabolic, cardiovascular, and neuroendocrine systems^1^,^2^,^3^. The administration of exogenous FGF21 can therapeutically correct the dysregulation with metabolic reversions of systemic metabolism, energy expenditure and insulin sensitivity^4^,^5^,^6^. Furthermore, the FGF21-resistance mediated by FGFRs-klb down-regulation in the liver and white adipose tissues proposed by Fisher and colleagues provides the explanation for the conditions that serum levels of the beneficial FGF21 are elevated in various metabolic diseases^7^,^8^.

It has been demonstrated that FGF21 can specifically bind to the membrane fibroblast growth factor receptors (FGFRs, FGFR1-4) that requires the expression of the trans-membrane protein β-klotho (klb) for signal transduction^9^. The biological signals of FGF21 are transduced by rapid phosphorylation of various downstream pathways, such as phosphorylation of Akt (protein kinase B) or extracellular mitogen-activated protein kinase 1 and 2 (ERK1/2) signaling pathway^9^,^10^,^11^. Moreover, the metabolic effects of FGF21 are functionally interplayed with the expression and activation of peroxisome proliferator-activated receptor-γ and -α (PPAR-γ/-α)^12^,^13^,^14^.

Hypertension (HTN) is a common complication of metabolic abnormalities in cardiovascular system^15^,^16^. As reported previously, serum FGF21 levels are closely associated with the metabolic syndrome and high blood pressure (hypertension)^1^,^17^. However, the direct downstream targets of FGF21 for the development of hypertension have not been revealed. It has been shown that FGF21 can cross the blood-brain barrier^18^, which raises a great possibility that FGF21 may act on the central nervous system^3^,^19^,^20^.

Notably, the baroreceptor reflex (baroreflex) afferent pathway, consisted of nucleus tractus solitarii (NTS) and nodose ganglion (NG), plays a key role in blood pressure modulation^21^,^22^,^23^,^24^. The defects in baroreflex function can lead to hypertension associated with metabolic syndrome and obesity^25^,^26^, which is consistent well with the notion that altered FGF21 receptor expressions may be observed in the NTS and NG^19^. Therefore, it is logically important to confirm and determine the role of FGF21 in the neurocontrol of blood pressure regulation and its contribution to the metabolic syndrome-related pathogenesis of hypertension.

In this study, we used the high fructose-drinking induced hypertension rats as a metabolic syndrome-related hypertension model^27^,^28^, and investigated the effects of FGF21 on blood pressure regulation and baroreflex sensitivity and the potential changes in expression profiles of FGFRs in baroreflex afferent pathway under disease condition.

## Results

### The Cardiovascular Effects of Chronic Intraperitoneal Infusion of Recombinant Human FGF21 in HFD Rats

Our preliminary data showed that the systolic blood pressure (SBP) and diastolic left ventricular internal diameter (LVIDd) were significantly increased in rats with four weeks high fructose-drinking (HFD, *n* = 42) compared to normal control (CTL, *n* = 8), rather than the body weight and the ratio of heart weight to body weight (HW/BW) (Figures S1 and 2).

**Figure 1:**
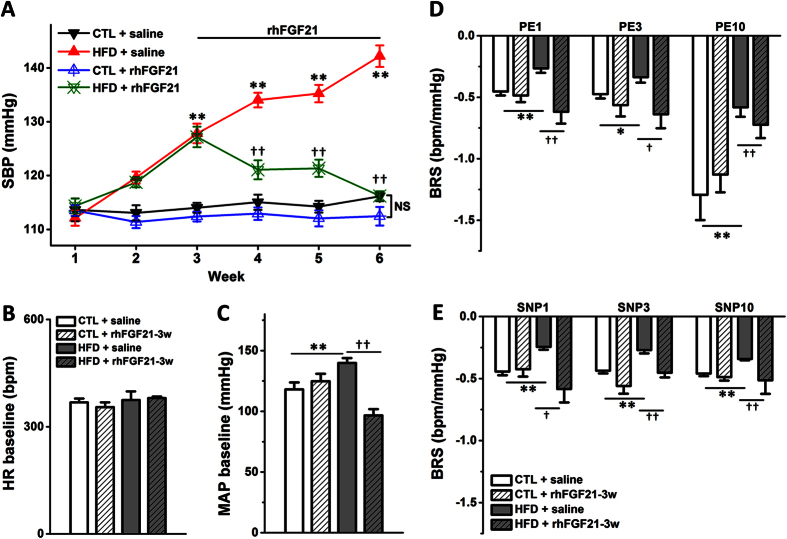
The cardiovascular protective effects of rhFGF21 administration (i.p) in HFD rats. (**A**) The SBP of control (CTL) and HFD rats with rhFGF21 infusion (i.p) for 3 weeks (3w), *n* = 8 for each group; (**B,C**) The MAP (mmHg) and heart rate (HR) baselines; (**D,E**) The BRS (bpm/mmHg) induced by PE or SNP-1/3/10 (phenylephrine or sodium nitroprusside at concentration of 1, 3 or 10 μg/kg). *n* = 4–8 for each group in (**B–E**). Results were analyzed using two-way ANOVA followed by Bonferroni’s *post hoc* test and averaged data were presented as mean ± SEM. *P < 0.05 and **P < 0.01 *vs.* CTL + saline, ^†^*P* < 0.05 and ^††^*P* < 0.01 *vs.* HFD + saline.

Based on this fructose fed induced hypertensive model, we investigated the cardiovascular effects by chronic intraperitoneal infusions of recombinant human FGF21 (rhFGF21) in HFD rats. As shown in Fig. 1A, the SBP of HFD rats treated with rhFGF21 (HFD + rhFGF21) was significantly and continuously decreased along with rhFGF21 infusion (5 μg/kg/day) during week 4, 5, and 6 compared to HFD groups (*P* < 0.01, respectively; *n* = 8 for each group). Meanwhile, significant modification of SBP was not observed in normal control rats under the same experimental condition (CTL + saline *vs.* CTL, *P* > 0.05, *n* = 8 for each group) (Fig. 1A).

Furthermore, we tested the mean arterial pressure (MAP, mmHg), heart rate (HR, bpm) and baroreflex sensitivity (BRS = ΔHR/ΔMAP, bpm/mmHg) of anesthetized rats by the protocol reported previously^1^. The reversal effect of rhFGF21 on the increased blood pressure in the HFD rats was also demonstrated by the notion of MAP baselines: HFD *vs.* CTL (*n* = 6–8) and HFD + rhFGF21-3w *vs.* HFD (*n* = 4–6, *P* < 0.01) (Fig. 1C). Meanwhile rhFGF21 infusions did not alter the HR baselines in the control or HFD rats (*P* > 0.05, *n* = 4–8) (Fig. 1B). Importantly, we further investigated the changes in BRS by means of intravenous injections of phenylephrine (PE) or sodium nitroprusside (SNP) at concentrations of 1, 3, or 10 μg/kg (PE1/3/10 or SNP1/3/10) in HFD and rhFGF21 treated rats. Our data demonstrated for the first time that the aberrantly decreased BRS in HFD group was significantly reversed by chronic rhFGF21 infusion (HFD + rhFGF21-3w *vs.* HFD: PE 1, 3, and 10, *P* < 0.05 or *P* < 0.01, respectively; SNP 1, 3 and 10, *P* < 0.05 or *P* < 0.01, respectively; *n* = 4–8) (Fig. 1D,E).

In addition, the echocardiographic results in the parasternal long axis (PSLA) M-mode indicated that the enlarged LVIDd in the HFD rats (6.66 ± 0.09 mm) was reduced obviously at 3 weeks after rhFGF21 infusion (5.91 ± 0.17 mm) (Table 1).

To investigate the status of metabolism and sympathetic nervous system in rats, we tested the serum levels of insulin, and norepinephrine as an indirect but widely used indicator of sympathetic nerve activity (SNA)^29^,^30^. Our data from ELISA(enzyme linked immunosorbent assay) showed that the markedly elevated serum insulin and norepinephrine in HFD, were reversed in HFD + rhFGF21-3w group (*n* = 8) (Figure S3). Interestingly, the serum insulin was also significantly up-regulated in CTL + rhFGF21-3w compared to CTL group (*n* = 8) (Figure S3). The results above suggested the protective effects of chronic FGF21 infusion on systemic hyperinsulinemia and excessive sympathoactivation in HFD.

### NTS and NG are Novel Targets for the Action of FGF21

**Figure 2:**
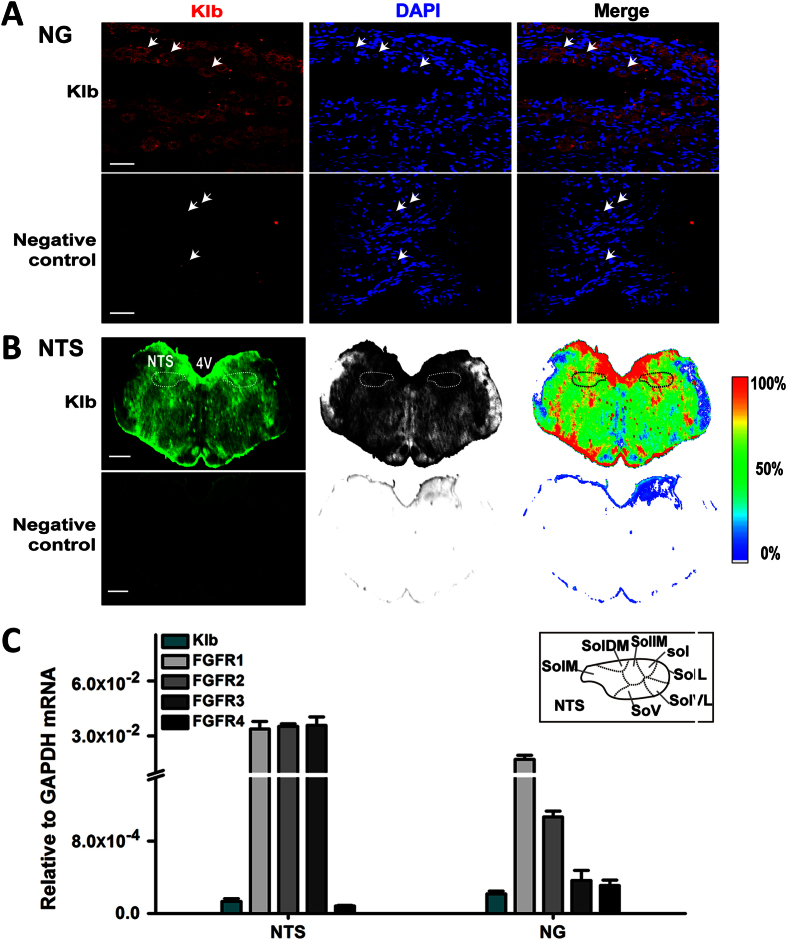
The distribution of Klb protein on the NG and NTS. (**A**) Klb protein was detected by immunostaining of NG tissues sections (7 μm). DAPI staining indicates nuclei (blue). The scale bar represents 50 μm, *n* = 8 duplications. (**B**) The immunostaining of whole brainstem section (35 μm; Bregma, −12.60 mm) showed the distribution of klb protein on NTS regions, *n* = 6 duplications, the scale bar represents 2 mm. (**C**) The mRNA expression levels of Klb and FGFR1-4 in the NTS and NG of normal rats, *n* = 3–6 duplications. The condition with no primary antibody was set as the negative control. 4V, 4th ventricle; sol, solitary tract; SolL nucleus of the solitary tract (Sol), -lateral part; SolVL, -ventrolateral part; SolV, -ventral part; SolM, -medial part; SolDM, -dorsomedial part; SolIM, -intermediate part.

To determine the direct targets of FGF21 on blood pressure control through baroreflex afferent function, we firstly investigated the mRNA and protein expressions of klb in the NTS and NG (Fig. 2). Our results from immunohistochemistry and whole section visualization showed that klb significantly expressed on the cell membrane of NG neurons, *n* = 8 duplications (Fig. 2A) and NTS regions, *n* = 6 duplications (Bregma −12.60 mm, the 6th edition, Paxinos and Watson, 2007) (Fig. 2B). Meanwhile, the Klb peptide blocking dot blot assay was performed to confirm the specificity of Klb protein immunostaining in NTS with LI-COR fluorescent-imaging (Figure S4). Furthermore, the mRNA expressions of Klb and FGFR1-4 in the NTS and NG tissues were confirmed by qRT-PCR detection (*n* = 3–6 duplications) (Fig. 2C).

### The Direct Effect of FGF21 on the Modifications of BRS Associated with the Signaling Activation in the NTS and NG.

**Figure 3:**
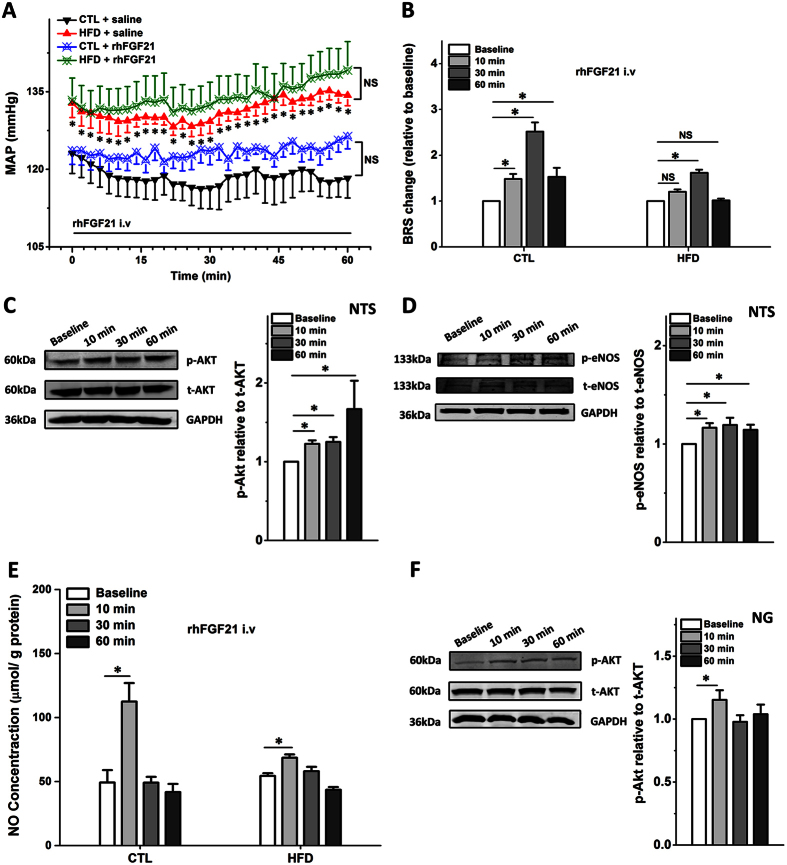
The acute intravenous rhFGF21 injections in HFD rats. (**A**) The MAP after rhFGF21 injection (i.v) within 1 hour, *n* = 5 for each group. (**B**) The BRS (PE, 3 μg/kg) change relative to basline (fold) at 10, 30 and 60 min after rhFGF21 injection, *n* = 5–6 for each group. (**C**) The Akt signal activation in the NTS at 10, 30 and 60 min after the rhFGF21 injections (i.v), *n* = 3–4 duplications from 12–15 rats for each group. (**D**) The eNOS signal activation in the NTS, *n* = 4 duplications from 12–16 rats for each group. (**E**) The nitric oxide (NO) concentrations in the NTS (*n* = 8 for each group). (**F**) The Akt signal activation in the NG at 10, 30 and 60 min after the rhFGF21 injections (i.v), *n* = 3–4 duplications from 12–15 rats for each group. p-, phosphorylated; t-, total. Results were analyzed using two-way ANOVA followed by Bonferroni’s *post hoc* test, and averaged data were presented as mean ± SEM. NS, not significant, *P < 0.05 *vs.* baseline. Gels have been cropped for clarity; the bands were confirmed by the comparison with full-length gel images (Supplementary Figure S7) and molecular weight.

The results above indicated that NTS and NG (baroreflex afferent pathway) are potential targets for the FGF21 action on neurocontrol of circulation (Figs 1 and 2), whereas, it is essential to confirm the direct effect of the FGF21 on the BRS control associated with baroreflex afferent pathway. To test this hypothesis, we administered rhFGF21 (1 μg/rats) intravenously (i.v) to rats, and continuously monitored the MAP for one hour under a stable anesthesia (3% amobarbital sodium, 25 mg/kg, i.p). Our data indicated that the MAPs were not significantly changed in normal control or HFD rats under this condition (Fig. 3A) (HFD + rhFGF21 *vs.* HFD, *P* > 0.05; CTL + rhFGF21 *vs.* CTL, *P* > 0.05, *n* = 5 for each group). As in the traditional protocol, the BRS of rats was usually tested under stable anesthesia^28^,^31^,^32^. In order to exclude the possible effect of amobarbital sodium on blood pressure under current situation, an equivelent volume saline was applied at 10, 30, and 60 min (Figure S5) in normal rats and the stable BRS conditions were achieved in anesthesized rats with 3% amobarbital sodium (25 mg/kg, i.p), suggesting that the applied dosage of amobarbital sodium would not influence the following tests with rhFGF21 on blood pressure. Appearently, our results indicated that the BRS was dramatically elevated by acute rhFG2F1 injections (i.v). In normal control rats, the BRS (relative to basline, fold) was 1.48 ± 0.11, 2.51 ± 0.20, and 1.53 ± 0.19 fold, respectivily, at 10, 30, and 60 min after rhFGF21 injections (*P* < 0.05, *n* = 5 for each group). While, in HFD rats, the increased BRS was only observed at 30 min (1.61 ± 0.07 *vs.* basline, *P* < 0.05, *n* = 5) after rhFGF21 treatment (Fig. 3B). In addition, the changes in ΔHR were similar to BRS (Figure S6).

Previous studies demonstrated that the Akt-eNOS(endothelial nitric oxide synthase)-NO(nitric oxide) pathway is the key signaling in blood pressure control in the NTS^33^, while Akt is also an important target of FGF21. Thus, to invesitigate the actions of acute rhFGF21 (i.v) infusion on NTS and NG, the Akt-eNOS-NO activations were also tested at the same time period. Our data showed that the phosphorylated levels of Akt and eNOS were significantly up-regulated compared with baselines at 10, 30, and 60 min in the NTS, and at 10 min in NG after acute rhFGF21 injections (i.v) in normal control rats (Fig. 3). In accordance with the Akt-eNOS signaling activation, the NO levels in the NTS at 10 min after acute rhFGF21 infusion were significantly elevated 2.28 fold in normal control rats, and 1.26 fold in HFD rats (Fig. 3). Whereares, the hypertensive agent superoxide (SO) in the NTS didn’t changed with the actions of rhFGF21 in normal control or HFD (Figure S6). Interestingly, the serum insulin was increased in normal control rats only after 60 min actions of FGF21, but didn’t changed in HFD rats, while the serum norepniphrine reducted only after 60 min of rhFGF21 action in HFD rats, but didn’t changed in normal control rats (Figure S3).

### The Expression of FGF21 and Its Receptors in the Liver of HFD Rats

**Figure 4:**
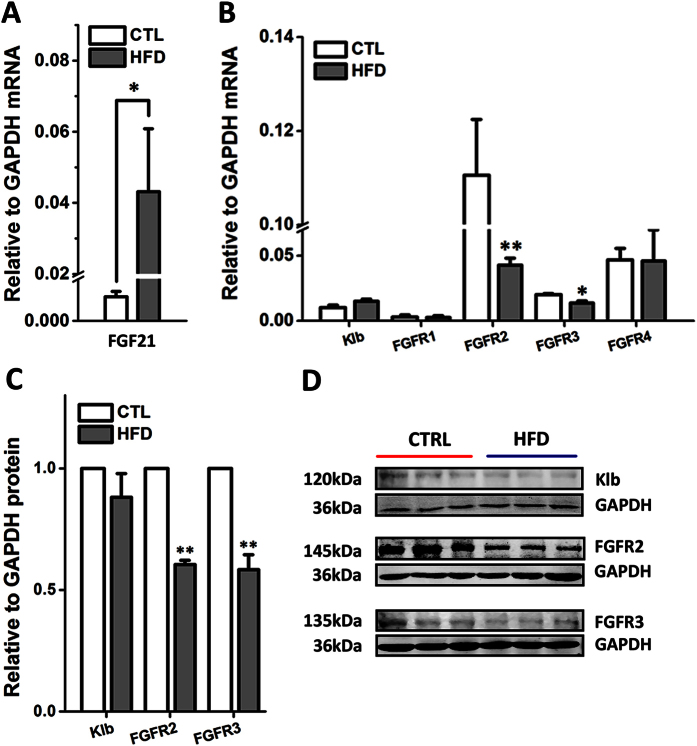
The mRNA and protein levels in the liver of HFD rats. (**A**) The mRNA expression of FGF21 in the liver of HFD (*n* = 9) and normal control rats (*n* = 6). (**B**) The FGFR1–4 and klb mRNA levels (*n* = 3–6). (**C**) The protein levels of klb, FGFR2 and FGFR3 protein (*n* = 6). Results were analyzed using two-tailed unpaired Student’s *t*-test, and averaged data were presented as mean ± SEM. *P < 0.05 and **P < 0.01 *vs.* CTL. The gels have been run under the same experimental conditions. Full-length blots were presented in Supplementary (Figure S7).

The dysregulation of FGF21 and its receptors in the liver were reported previously in obesity and insulin resistant rats^7^. The results above demonstrated the cardiovascular beneficial effects and the modifications of baroreflex afferent function by rhFGF21 administration in HFD rats, while the pathological status of FGF21 in HFD rats is unrevealed. In this study, our data showed an approximate 25 folds higher of FGF21 mRNA expression in the liver in HFD rats (*n* = 9) compared to control rats (*n* = 6, *P* < 0.05) (Fig. 4A). Moreover, the mRNA and protein levels of FGFR2 (*P* < 0.01, *n* = 6 for mRNA and protein) and FGFR3 (*P* < 0.05, *n* = 3 for mRNA and *P* < 0.01, *n* = 6 for protein) were significantly decreased in HFD groups (Fig. 4B,C), strongly suggesting that the HFD rats underwent a clear FGF21-resistant condition as reported previously^7^,^8^.

### The Dysregulation of FGF21 Receptors in Baroreflex Afferent Pathway in HFD Rats

The effects of FGF21 are dependent on the expression levels of its receptors^7^,^8^. Thus, it was essential to further investigate the expression of klb and FGFR1-4 in the baroreflex afferent pathway in HFD rats.

**Figure 5:**
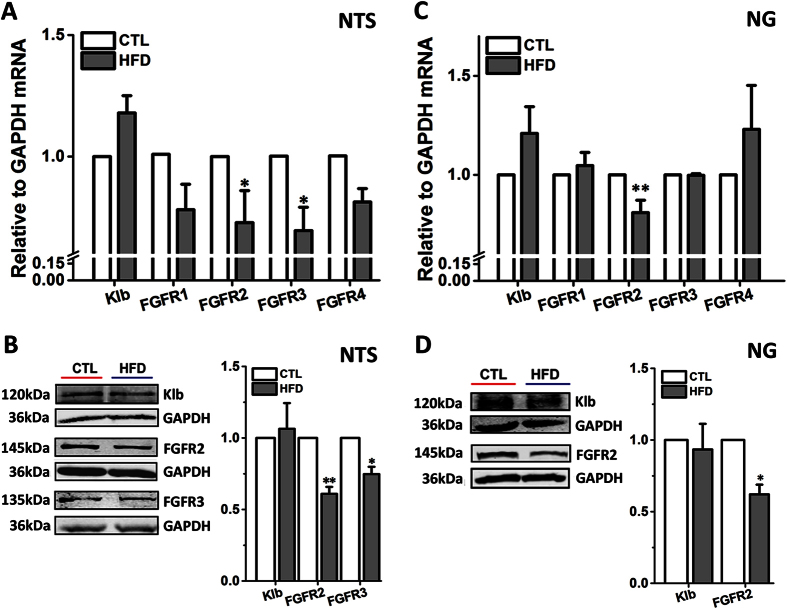
The dysregulation of FGFRs-Klb at mRNA and protein levels in the NTS and NG in HFD rats. (**A**) The klb and FGFR1–4 mRNA levels in the NTS tissues of HFD and control group rats (*n* = 6–9 duplications from 18–27 rats for each group). (**B**) The protein levels for klb (*n* = 3 duplications from 12–15 rats for each group), FGFR2 (*n* = 6 duplications from 24–30 rats for each group) and FGFR3 (*n* = 4 duplications from 14–20 rats for each group) in NTS. (**C**) The klb and FGFR1–4 mRNA levels in the NG tissues of HFD and control group rats (*n* = 5 duplications from 20–25 rats for each group). (**D**) The klb and FGFR2 protein expression in the NG (*n* = 3 duplications from 15–18 rats for each group). Results were analyzed using two-tailed unpaired Student’s *t*-test, and averaged data were presented as mean ± SEM. *P < 0.05 and **P < 0.01 *vs.* CTL. The gels have been run under the same experimental conditions. Full-length blots were presented in Supplementary (Figure S7).

In the NTS tissues, results from qRT-PCR showed the significant mRNA down-regulations of both FGFR2 and FGFR3 (*P* < 0.05, *n* = 7 and 9 duplications, respectively), while the similar alternations were not confirmed for klb, FGFR1, and FGFR4 in HFD model rats (Fig. 5A). The protein detection results were consistent well with the changes in mRNA levels. The difference of klb protein expression was also not observed in HFD group (*P* > 0.05, *n* = 3 duplications) (Fig. 5B), but the proteins for FGFR2 and FGFR3 significantly decreased (*P* < 0.01, *n* = 6 duplications; *P* < 0.05, *n* = 4 duplications, respectively).

In the NG tissues, the mRNA (*P* < 0.01, *n* = 5 duplications) and protein (*P* < 0.05, *n* = 3 duplications) expressions of FGFR2 were significantly down-regulated; but the mRNA and protein levels of klb, and mRNA levels of FGFR 1, 3, and 4 were normal (Fig. 5C,D).

### The Genetic Modifications of PPAR-γ and -α in the NTS and NG by Chronic rhFG2F1 Infusion in HFD Rats.

**Figure 6:**
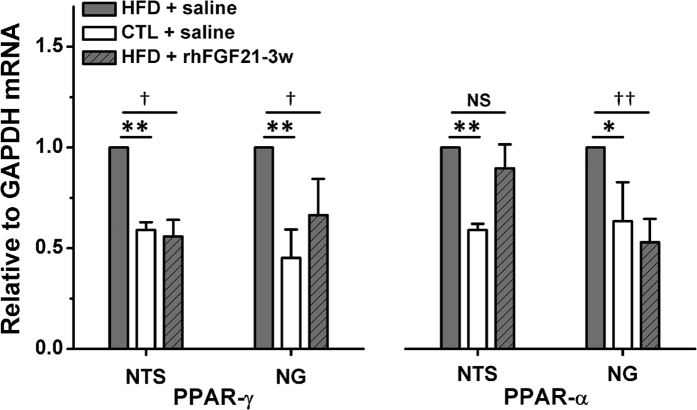
The expression of PPAR-γ and -α in the NTS and NG in chronic rhFGF21 infusion rats. The mRNA levels of PPAR-γ and -α in the NTS and NG in HFD + saline, CTL + saline and HFD + rhFGF21–3w (5 μg/kg/day, 3 weeks, i.p) rats. *n* = 4 duplications from 8–12 rats for each group. Results were analyzed using one-way ANOVA followed by Bonferroni’s *post hoc* test, and averaged data were presented as mean ± SEM. NS, not significant, *P < 0.05 and **P < 0.01 *vs.* CTL + saline; ^†^*P* < 0.05 and ^††^*P* < 0.01 *vs.* HFD + saline.

The PPAR-γ and -α are closely associated with the actions of FGF21 in various biological functions^12^,^13^,^14^. Our results demonstrated that the mRNA levels of PPAR-γ and -α in the NTS and NG significantly up-regulated in HFD rats (HFD + saline *vs.* CTL saline, NTS: *P* < 0.01 for PPAR-γ and -α; NG: *P* < 0.01 for PPAR-γ and *P* < 0.05 for PPAR-α) (Fig. 6). Meanwhile, the chronic rhFGF21 infusion (5 μg/kg/day) significantly reversed the increased levels of PPAR-γ and -α (HFD + saline *vs.* HFD + rhFGF21-3w, NTS: *P* < 0.05 for PPAR-γ, while *P* > 0.05 for PPAR-α; NG: *P* < 0.05 for PPAR-γ and *P* < 0.01 for PPAR-α, *n* = 4 duplications from 8–12 rats for each group) (Fig. 6).

## Discussion

The high fructose treated rats used in this research showed significant hypertension with impaired BRS, hyperinsulinemia and elevated SNA (serum NE increased). Meahwhile, the pharmacotherapeutic experiments *in vivo* have revealed the beneficial effects on blood pressure (SBP and MAP) and BRS in HFD rats by three weeks chronic intraperitoneal rhFGF21 infusion (Fig. 1). In addition, the markedly increased SBP (afterload) and LVIDd (preload) in HFD, were both reduced by chronic rhFGF21 infusion. Therefore, the reduction of SBP and LVIDd by chronic rhFGF21 treatment may be induced by its effects on sympathoinbition (SNA/serum NE decreased) and BRS improvement.

For a long time, the direct targets and mechanisms of blood pressure control by FGF21 actions haven’t been reported, and it may be contributed by its effects on metabolism (hyperinsulinemia) and/or actions on baroreflex and sympathetic nerve. Whereas, our data in this study fully supported our hypothesis by the notion of the functional expressions of klb and FGFRs proteins in the NG and NTS regions (Figs 2 and 5), and these results are the major findings of the current investigation and fundamental base leading to the investigation on the roles of FGF21 in the neurocontrol of circulation and blood pressure regulation. FGF21 ameliorated the hypertension and BRS impairment in HFD, which may be mediated by its receptors located in baroreflex afferent pathway.

Of note, the BRS (ΔHR/ΔMAP) represents the function and ability of baroreflex to control blood pressure, and it may contribute to the modifications on the changes in blood pressure and heart rate^31^,^32^,^34^, therefore, the increase of BRS actually doesn’t mean the activation of baroreflex or sympathoactivation. In this study, despite no alteration was observed for the MAP by an acute intravenous rhFGF21 injections in control and HFD rats, the BRS and ΔHR were increased within one hour, with ΔMAP didn’t changed (Figs 3 and S6). Thus, the systemic rhFGF21 administration may cause complex effects on cardiovascular system in acute injections with BRS improvement, and it may not have direct and acute effects on the blood pressure regulation as other vasoactive drugs. Whereas, the Akt signaling is an important downstream target of the FGF21 actions in the brain neurons for the neuroprotective effects^20^, moreover, the Akt-eNOS signaling was demonstrated as a central pathway for baroreflex and blood pressure control in the NTS^35^,^36^. We demonstrated that the rapid and significant phosphorylation of Akt-eNOS, and the increase of NO level in the NTS were induced by acute injection of rhFGF21 within one hour (Fig. 3). In addtion, the Akt-eNOS-NO signaling pathway in the NTS has been widely reported to be involved in the neuroexcitation modification or blood pressure regulation^35^,^36^, therefore, the obviously up-regulated BRS by acute rhFGF21 infusion in rats, is partially related to the activation of Akt-eNOS-NO pathway in the NTS (Fig. 3), but independent to the levels of serum insulin and superoxide in the NTS (Figures S3 and S6).

The metabolic effects of FGF21 are functional interplayed with the activation of PPAR-γ/-α^12^,^13^,^14^. In addition, the previous reports demonstrated that the PPAR-γ was dysregulated in nodose ganglion neurons or rostral ventrolateral medulla (RVLM) neurons in high fat diet or spontaneously hypertensive rats, respectively^37^,^38^. Furthermore, PPARs contribute to the blood pressure control via the central nervous system by the modifications of oxidative stress in neurons^38^. In this study, we demonstrated that the aberrantly up-regulated PPAR-γ and -α in the NTS and NG of HFD rats were markedly reversed by chronic rhFGF21 infusion (Fig. 6). Thus, the PPAR-γ and -α pathway in the NTS and NG may be involved in the protective effects of rhFGF21 on the increased SBP and impaired BRS in HFD rats.

FGF21-resistance mediated by FGFRs-klb down-regulation in the peripheral metabolic and hypothalamus tissues was demonstrated recently^7^,^8^. In our study, the FGFRs down-regulation in the NG and NTS regions of the HFD rats is partially indicated by the expression profiles of mRNA and protein in the current observations (Fig. 5). Meanwhile, the up-regulated NO level in the NTS by acute rhFGF21 injections induced Akt-eNOS signaling activations, was only 1.26 fold in HFD, which was lower than 2.28 fold in normal control rats (Fig. 3). Moreover, the FGF21-resistant status has also been demonstrated in the liver tissues, which is supported by the notion that the FGF21 mRNA is markedly up-regulated, while FGFR2 and FGFR3 mRNA and protein are significantly down-regulated in the HFD rats (Fig. 4). Therefore, the FGF21-resistance in baroreflex afferent pathway might also be an important aspect to understand the pathogenesis of the metabolic disorder related hypertension, and it is attractive to investigate the role of FGF21 on hypertension by receptors (klb-FGFRs) knockdown/knockout in the NTS or NG in further investigation.

FGF21 can penetrate the blood brain barrier to act on the central nervous system (CNS)^3^,^18^. Previous studies have demonstrated the regulatory effects on neuroendocrine, neuroprotection and central metabolism control induced by FGF21 actions on hypothalamus and other CNS areas^3^,^19^,^20^. Recently, it was revealed that the stimulation of SNA to brown adipose tissue was induced by the actions of FGF21 in brain, depending on the corticotropin-releasing factor^39^. Whereas, the corticotropin-releasing factor depended sympathoactivation induced by FGF21 or leptin only regulated its thermogenic functions, but didn’t modificated on cardiovascular actions^39^,^40^. Thus, the autonomic functions by central actions of FGF21 is not clarified well and still needs further research. Even though, our immunohistochemistry suggested that klb may widely distributed in brainstem (immuno-positive areas), but whether the FGF21 could act on other autonomic function areas in CNS also needs further evidences to demonstrated in future, such as FGF21 receptors expressions, signaling activations and functions.

On the basis of above results, it is attractive to investigate the role of FGF21 on hypertension by gene knockdown or knockout of FGF21 receptors (klb-FGFRs) in the NTS and NG in further investigation. Whereas, the findings of this study indicated that the NTS and NG are the novel and direct targets of the central FGF21 action for the blood pressure control associated with baroreflex afferent pathway.

## Methods

### Animals

Male Sprague-Dawley (SD) rats weighting 200–220 g were purchased from the experimental animal center of the Second Affiliated Hospital of Harbin Medical University (Harbin, China).

All animal protocols were pre-approved by the Institutional Animal Care and Use Committee at Harbin Medical University, which are in accordance with the recommendations of the Panel on Euthanasia of the American Veterinary Medical Association and the National Institutes of Health publication “*Guide for the Care and Use of Laboratory Animals*”.

### High fructose-drinking induced Hypertension in Rats

Rats were randomly housed in groups of 3–4 rats per cage in a controlled environments (25 °C with 12 hours light/dark cycles). According to the previous reports^27^,^41^, rats recevied standard rat chow and tap water (Control group, CTL), or water containing 10% (weight/volume) fructose (high fructose-drinking group, HFD) for 4–5 weeks. The systolic blood pressure of rats for each group was recorded weekly.

### Systolic Blood Pressure Measurements

The non-invasive systolic blood pressure (SBP, mmHg) of all rats were measured weekly. The SBP was measured in conscious rats with a manometer-tachometer (BP-2010E, Softeron Biotechnology, Beijing, China) using the tail-cuff method. Rats were placed in a plastic holder under a 36 °C temperature-controlled quiet environment. The average value of SBP for each rat was obtained from five SBP readings after the rats were acclimated to the environment.

### Echocardiographic Measurements

Trans-thoracic echocardiography with an ultrasound machine (Vevo 2100 imaging system, VisualSonics, Toronto, Canada) was used to test the heart functions of rats. Left ventricular systolic/diastolic internal diameter (LVIDs/LVIDd, mm), interventricular septum systolic/diastolic thickness (IVSs/IVSd, mm) and Left ventricular systolic/diastolic posterior wall (LVPWs/LVPWd, mm) were measured, and ejection fraction (EF, %) and fractional shortening (FS, %) were calculated from the short axis (SAX) or parasternal long axis (PSLAX)-mode recording.

### Baroreflex Sensitivity Detection

By following the protocol^31^,^32^, the mean arterial pressure (MAP, mmHg), heart rate (HR, bpm) and baroreflex sensitivity (BRS) of anesthetized (3% amobarbital sodium, 25 mg/kg, i.p) rats were tested by cannulas of arteria (left, for artery pressure detection) and vena (right, for drug administration) femoralis and electrocardiographic (ECG) recording (LabChart 7 Pro software, ADinstruments, Australia) with body temperature maintained at approximately 35 °C. BRS was established by intravenous bolus injections of phenylephrine (PE, Sigma, USA) or sodium nitroprusside (SNP, Sigma, USA) at an incremental dose (1 μg/kg, 3 μg/kg and 10 μg/kg), respectively, and 15 to 20 minutes was given before the next injection. The maximum changes in HR and the associated MAP were calculated as BRS (ΔHR/ΔMAP, bpm/mmHg).

### Chronic intraperitoneal infusions of rhFGF21 in HFD Rats

Rats were subjected to intraperitoneal infusions of 0.9% sterile saline (HFD + saline) or rhFGF21 (5 μg/kg/day, HFD + rhFG2F1) for 3 weeks after 2 weeks of high fructose-drinking, and the access to fructose-drinking continued. Meanwhile, the control rats were subjected to infusions of 0.9% sterile saline (CTL + saline) or rhFGF21 (5 μg/kg/day, CTL + rhFG2F1) at the same time. The rhFGF21 (CYT-281) was purchased from ProSpecBio (East Brunswick, NJ, USA).

### Acute intravenous rhFGF21 injections in HFD rats

After 4 weeks of high fructose-drinking or normal fed, the anesthetized rats were subjected to femoral artery and vein catheters to record mean arterial pressure (MAP), and injects drug, respectively. After intravenous rhFG2F1 (1 μg/rats) or 0.9% sterile saline injections, MAP (mmHg) was monitored continuously for one hour. Meanwhile, the BRSs (ΔHR/ΔMAP, bpm/mmHg) induced by phenylephrine (PE, 3 μg/kg) were detected before (BRS basline) or after injections (at 10, 30 and 60 min) in HFD and control rats.

### Preparation of tissues

The nodose ganglia and liver tissues were isolated from each group rats as previously described by our laboratory method^24^,^42^; meanwhile the bilateral NTS were dissected with 1 mm inner diameter from a brainstem section (1 mm thick) at the level of the obex under a microscope^43^.

### Quantitative Real-Time Polymerase Chain Reaction (qRT-PCR)

One mRNA sample of NG or NTS tissues was extracted from 4–5 rats in the same group (control or HFD). All primers used see the Table S1. The mRNA expression was determined using SYBR Green reagent in ABI 7500 Real-Time PCR System (Applied Biosystems). Data of relative gene expression were analysed with 2^−ΔΔCT^ method^44^.

### Immunoblotting Analysis

Total proteins were prepared by homogenizing the isolated NTS, NG or liver tissues for 1 hour at 4 °C in RIPA buffer containing Protease Inhibitor Cocktail. One protein sample of NG or NTS tissues was extracted from 4–5 rats in the same group. Protein extracts (100 μg/sample, accessed through a BCA protein assay) were subjected to 10% SDS-Tris glycine gel electrophoresis and then transferred (Bio-Red Laboratories, USA) to a nitrocellulose membrane. The membranes were blocked in 5% non-fat dry milk/PBS buffer for 2 hours, and then incubated at 4 °C overnight with the primary antibodies (1:200–1:500): anti-GAPDH (internal control, Sigma, USA), anti-klb/anti-FGFR3 (Santa Cruz, USA), anti-FGFR1/anti-FGFR4 (Sigma, USA), anti-FGFR2 (Abcam, USA), anti-p-Akt/anti-t-Akt and anti-p-eNOS/anti-t-eNOS (Cell Signaling Technology, USA), then the appropriate secondary anti-bodies (1:8000; LI-COR Biosciences, Lincoln, NE) were used at room- temperature for 1 hour. The results were detected and analysed via odyssey system (LI-COR Biosciences, Lincoln, NE).

### Measurements of Nitric Oxide and Superoxide Levels in the NTS

The levels of nitric oxide and superoxide anion in isolated NTS tissues were measured using Nitric Oxide kit (Griess reagent, S0021) and Superoxide assay kit (WST-1, S0060), respectively (Beyotime institute of Biotechnology, China).

### Enzyme Linked Immunosorbent Assay(ELISA)

The serum insulin and norepinephrine concentrations of rats were measured by using ELISA kits (E-EL-R2466c and E-EL-0047c, respectively; Elabscience, China).

### Immunohistochemistry and Whole Section Visualization

The immunohistochemistry protocol for NG was described by our previous reports^24^,^45^: Horizontal sections of 7 μm thickness were collected for later klb protein staining (anti-klb, 1:100, Santa Cruz, USA).

Whereas, for the whole section visualization of NTS^46^, rats were anesthetized with 3% amobarbital sodium (25 mg/kg, i.p), then transcardially perfused for 10 minutes with 4% paraformaldehyde (PFA). Harvested brains were placed in 4% PFA overnight before transferred to 30% sucrose and rotated for 24~48 hours at 4 °C. Coronal brainstem sections (35 μm thickness, Bregma −12.6 mm) were cut on a freezing microtome (Leica Biosystem, Germany) and stored in −80 °C. Brainstem sections were washed in PBS for 10 minutes each before immunostaining, and blocked in 4% normal goat serum/0.3% Triton X-100/PBS for 1 hour at room temperature. For labeling, the sections were incubated with primary antibody overnight at 4 °C: anti-klb (Santa Cruz, USA) at a dilution of 1:200 in blocking solution. Then the sections were washed three times with PBST for 10 minutes each, and incubated in the appropriate secondary antibody (anti-goat IRDye 800CW) at a dilution of 1:5000 in PBS (LI-COR Biosciences, Lincoln, NE) at room temperature for 1 hour. All sections washed five times in PBST for 10 minutes each. And then fluorescent-imaging was detected using a LI-COR Odyssey infrared imager (21 μm resolution, 1 mm offset at the highest quality).

### Statistical Analysis

All data were presented as mean ± SEM. Statistically significant differences among more than two groups were analysed with one or two-way ANOVA followed by Bonferroni’s *post hoc* test, and the two-tailed unpaired Student’s *t*-test was used where is appropriate. The criterion for statistical significance was set at *P* < 0.05.

## Additional Information

**How to cite this article**: He, J.-L. *et al*. FGF21 ameliorates the neurocontrol of blood pressure in the high fructose-drinking rats. *Sci. Rep.* **6**, 29582; doi: 10.1038/srep29582 (2016).

## Supplementary Material

Supplementary Information

## Figures and Tables

**Table 1:** Cardiac Parameters of rhFGF21 Administration in HFD Rats.

PSLAX M-Model Parameters	CTL + saline (*n* = 9)	HFD + saline (*n* = 11)	HFD + rhFGF21-3w (*n* = 4)	CTL + rhFGF21-3w (*n* = 5)
IVSd (mm)	1.94 ± 0.10	1.76 ± 0.02	1.79 ± 0.08	2.07 ± 0.12
IVSs (mm)	3.08 ± 0.12	2.88 ± 0.06	2.86 ± 0.07	3.36 ± 0.19
LVIDd (mm)	6.15 ± 0.19	6.66 ± 0.09*	5.91 ± 0.17^†^	6.34 ± 0.24
LVIDs (mm)	3.07 ± 0.12	3.49 ± 0.19	2.95 ± 0.24	3.34 ± 0.22
LVPWd (mm)	1.99 ± 0.07	2.15 ± 0.07	1.76 ± 0.11	2.49 ± 0.25
LVPWs (mm)	3.18 ± 0.11	3.21 ± 0.14	2.74 ± 0.25	3.36 ± 0.01
EF (%)	79.6 ± 2.54	74.6 ± 2.37	79.8 ± 2.99	77.4 ± 2.77
FS (%)	49.7 ± 2.53	44.9 ± 2.33	50.3 ± 3.22	47.4 ± 2.63

All data are presented as mean ± SEM. PSLAX, parasternal long axis; LVIDs/LVIDd, systolic/diastolic left ventricular internal diameter, IVSs/IVSd, systolic/diastolic left ventricular septum thickness; LVPWs/LVPWd, systolic/diastolic left ventricular posterior wall; EF, ejection fraction; FS, fractional shortening. CTL: control rats; HFD: high fructose-drinking rats; rhFGF21-3w: intraperitoneal infusion of rhFGF21 for 3 weeks. Group sizes are as *n* animals. *P < 0.05 *vs.* CTL; ^†^*P* < 0.05 *vs.*HFD.
