# Supplementary material for: FGF21 ameliorates the neurocontrol of blood pressure in the high fructose-drinking rats
Source: Sci Rep. 2016 Jul 8;6:29582. doi: 10.1038/srep29582 (PMC4937430; doi:10.1038/srep29582)
Supplement: Supplementary Information [file srep29582-s1.pdf]

ONLINE SUPPLEMENTAL

FGF21 ameliorates the neurocontrol of blood pressure in the high fructose-drinking rats.

Jian-Li He [a,1], Miao Zhao [a,1], Jing-Jun Xia [b,1], Jian Guan [a], Yang Liu [a], Lu-Qi Wang [a], Dong-Xue Song [a], Mei-Yu Qu [a], Meng Zuo [a], Xin Wen [a], Xue Yu [a], Rong Huo [a], Zhen-Wei Pan [a], Tao Ban [a], Yan Zhang [a], Jiu-Xin Zhu [a], Weinian Shou [c], Guo-Fen Qiao [a,] *, Bai-Yan Li [a,] *

[a] Department of Pharmacology (State-Province Key Laboratories of Biomedicine-Pharmaceutics of China, Key Laboratory of Cardiovascular Medicine Research, Ministry of Education), College of Pharmacy, Harbin Medical University, Harbin, China

[b] Department of Orthopedics , the Second Affiliated Hospital of Harbin Medical University, Harbin, China

[c] Riley Heart Research Center, Division of Pediatric Cardiology, Herman B. Wells Center for Pediatric Research, Department of Pediatrics, Indiana University School of Medicine, Indianapolis, USA

*Corresponding Author: Bai-Yan Li and Guo-Fen Qiao, Department of Pharmacology, Harbin Medical University, #157 Bao-Jian Road, Harbin 150081, China; Phone/Fax: +86 451-86671354; E-mail: liby@ems.hrbmu.edu.cn (B.-Y. Li), or qiaogf88@163.com (G.-F. Qiao)

[1]These authors contributed equally to this work.

1. Table S1. All the primers used in this study.

Gene List	primers	Sequences
**GAPDH**	Sense primer	5'-ATGACTCTACCCACGGCAAG-3'
	Antisense primer	5'-TACTCAGCACCAGCATCACC-3'
**Klb**	Sense primer	5'-CAGAGAAGGAGGAGGTGAGG-3’
	Antisense primer	5'-CAGCACCTGCCTTAAGTTGA-3’
**FGFR1**	Sense primer	5'-TGGCACCTGAGGCATTGTT-3′
	Antisense primer	5'-AAGAGCACCCCAAAAGACCAC-3′
**FGFR2**	Sense primer	5'-ACCAACTGCACCAATGAACTGT-3′
	Antisense primer	5'-TTAAACGTGGGCCTCTGTGA-3′
**FGFR3**	Sense primer	5′-TGCCTGCTGACCCCAAGT-3′
	Antisense primer	5'-CCTGTCCAAAGCAGCCTTCT-3′
**FGFR4**	Sense primer	5′-CCGGCCAGACCAAACC-3′
	Antisense primer	5'-TCAGGTCTGCCAAATCCTTGT-3′
**PPAR-α**	Sense primer	5'-TGGTGGACCTCCGGCA-3'
	Antisense primer	5'-TCTTCTTGATGACCTGCACGA-3'
**PPAR-γ**	Sense primer	5'-ATTCTGGCCCACCAACTTCGG-3'
	Antisense primer	5'-TGGAAGCCTGATGCTTTATCCCCA-3'

2. Figure S1 - 6:

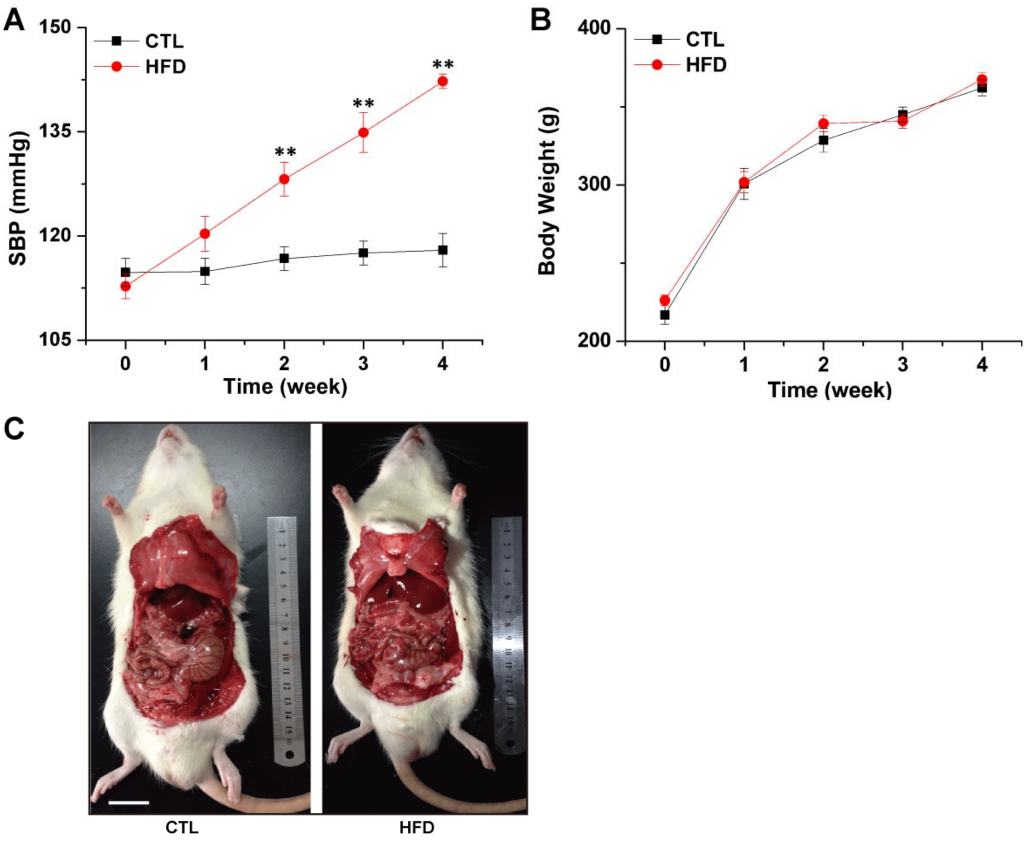

Figure S1. Dysfunction of the systolic blood pressure in HFD rats. (A-B) The SBP and body weight of HFD (n = 42) and parallel normal control (n = 8) rats were detected from week 0 to 4; **(C)** The photograph representing the gross anatomy of control and HFD rats. There was no intra-abdominal fat accumulation in HFD rats. The scale bar represents 2 cm. Results were analyzed using one-way ANOVA followed by Bonferroni's *post hoc* test and averaged data were presented as mean ± SEM. $^{**}P < 0.01$ *vs*. CTL.

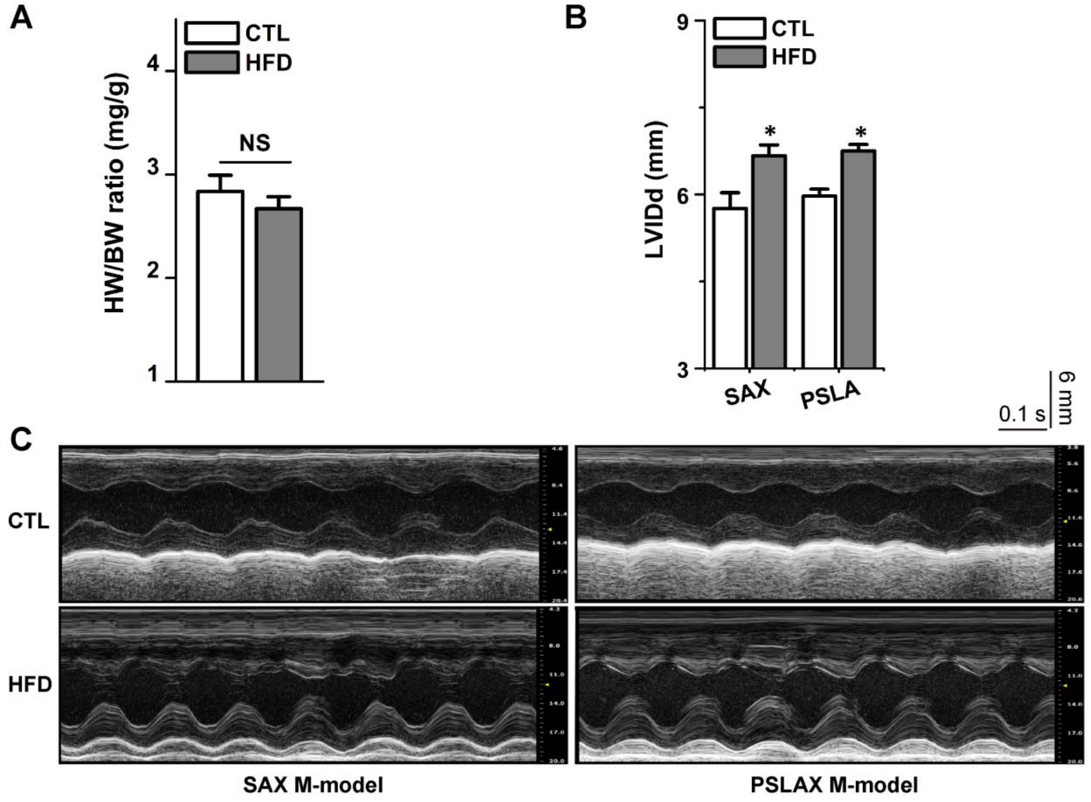

Figure S2. The cardiac parameters in HFD rats. **(A)** The ratio of heart weight to body weight (HW/BW, mg/g) of HFD and control rats (*n* = 4 for each group); **(B)** the diastolic LVID (LVIDd, mm) was calculated (*n* = 5 for each group); **(C)** The photograph representing the cardiac morphology of control and HFD rats on SAX- or PLSA-M model;. Results were analyzed using two-tailed unpaired Student's t-test and averaged data were presented as mean ± SEM. NS, not significant; *$P < 0.05$ *vs*. CTL.

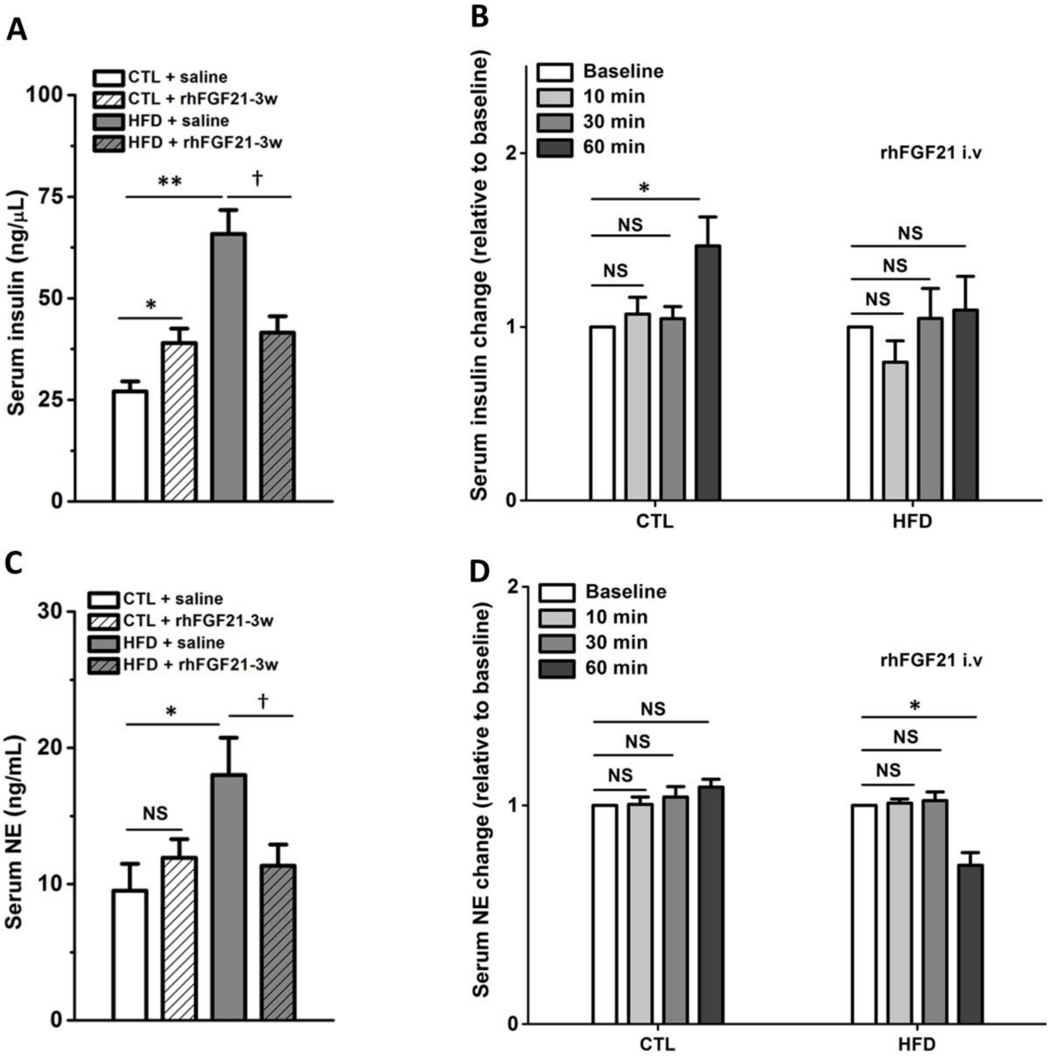

Figure S3. The serum insulin and norepinephrine in HFD and FGF21 treated rats. **(A-B)** The serum insulin levels, $n = 8$ for each group. **(C-D)** The serum norepinephrine (NE) levels, $n = 8$ for each group. Results were analyzed using two-way ANOVA followed by Bonferroni's *post hoc* test, and averaged data were presented as mean ± SEM. NS, not significant, $*P < 0.05$ and $**P < 0.01$ *vs.*CTL or baseline; $^{\dagger}P < 0.05$ *vs.* HFD + saline.

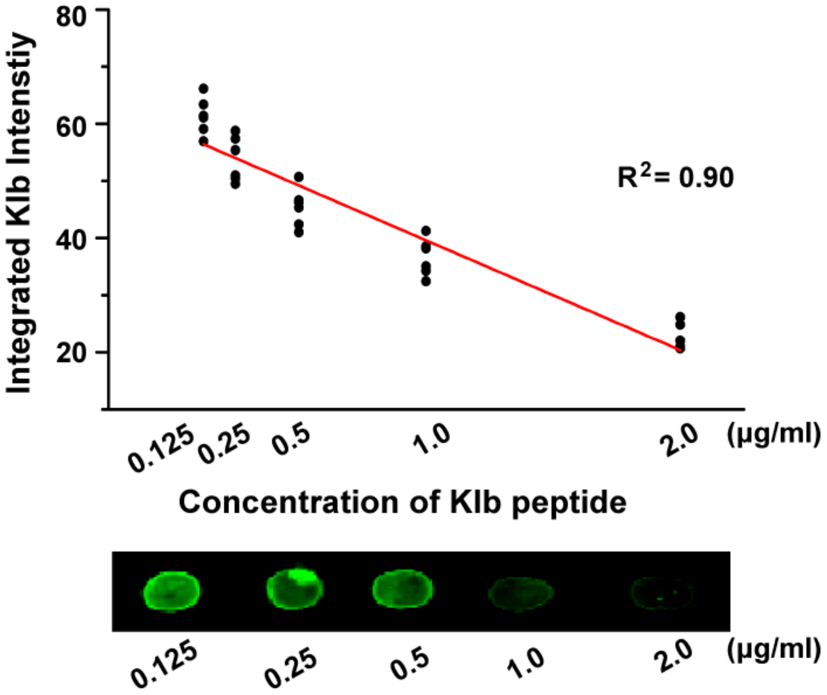

Figure S4. The Klb peptide blocking dot blot results using LI-COR. Total proteins of NTS tissues were dotted on a nitrocellulose membrane (dot-blot), *n* = 6. In accordance with the method of NTS immunostaining for klb protein, the membranes were incubated with a cocktail of primary antibody (anti-klb Santa Cruz) at a dilution of 1:200 and Klb peptide (Santa Cruz) with a decrease in the concentration (beginning with 2.0 μg/ml, and a serial dilution of 1:2). The dilution of secondary antibody was 1:5000. The fluorescent-imaging was detected using a LI-COR Odyssey infrared imager. The integrated intensities (green dot) caluculated from LI-COR Odyssey confirmed a reliable and negative linear relationship with the concentration of additional klb peptide. The Klb peptide specifically blocked the binding of primary antibody and Klb protein in the NTS.

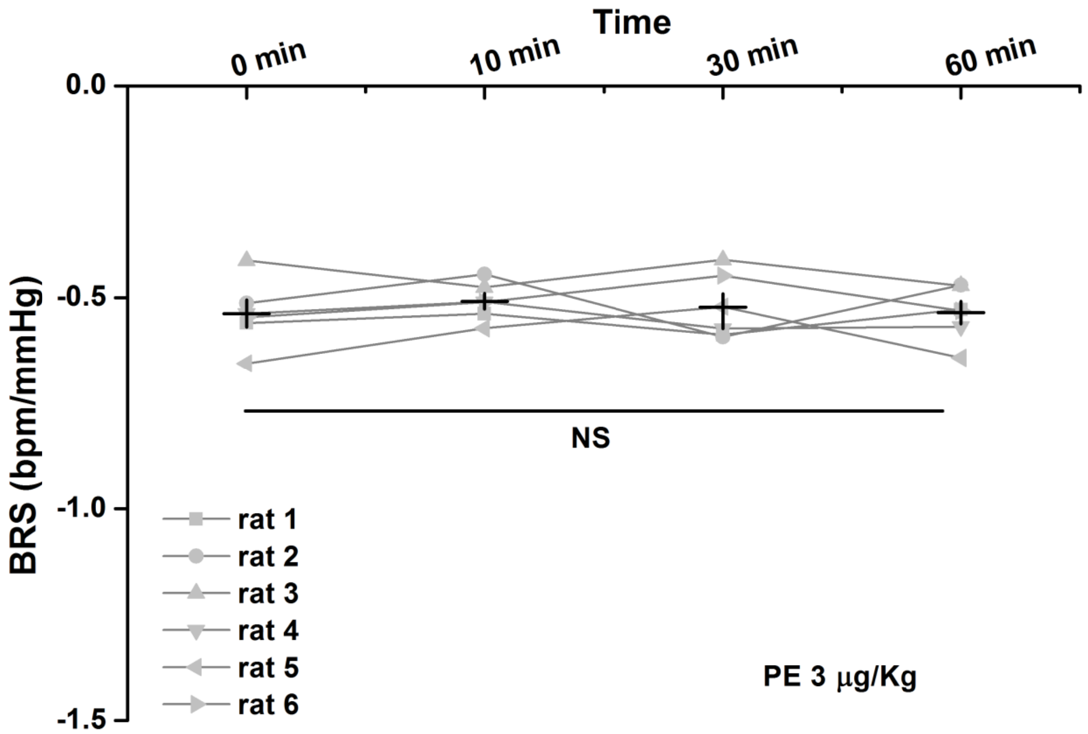

Figure S5. The stable BRS under anesthesia within one hour. The BRSs were detected (PE: 3 μg/Kg) after saline injection under a stable anesthesia (3% amobarbital sodium, 25 mg/kg, i.p) at 10, 30 and 60 min in normal control rats, *n* = 6 for each group. Results were analyzed using one-way ANOVA followed by Bonferroni's *post hoc* test, and averaged data were presented as mean ± SEM. NS, not significant, *vs.* baseline.

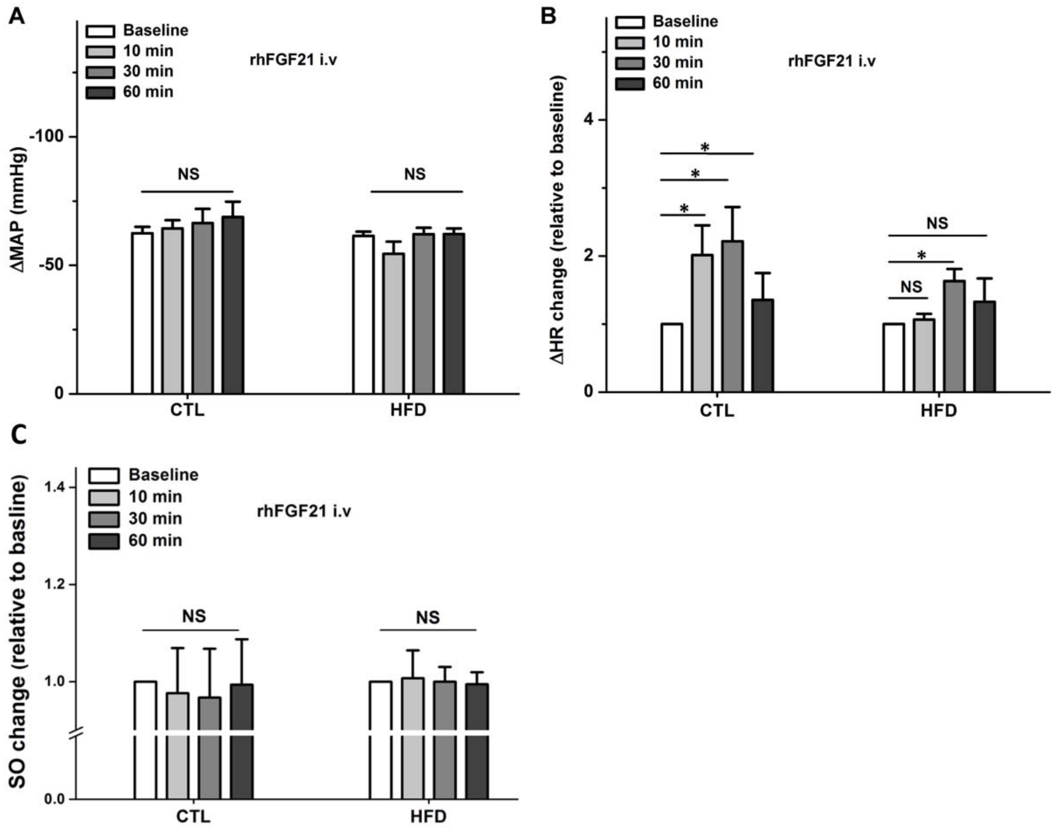

Figure S6. The ΔMAP, ΔHR and superoxide level in the NTS after acute rhFGF21 injections. **(A)** The ΔMAP (mmHg) induced by PE (3 μg/kg) at 10, 30 and 60 min after rhFGF21 injection. **(B)** The ΔHR(bpm) change relative to basline (fold), *n* = 5-6 for each group. **(C)** The superoxide (SO) level in the NTS at 10, 30 and 60 min after rhFGF21 injection, relative to basline (fold, OD value/ OD value), *n* = 8. Results were analyzed using two-way ANOVA followed by Bonferroni's *post hoc* test, and averaged data were presented as mean ± SEM. NS, not significant, *$P < 0.05$ *vs*. baseline.

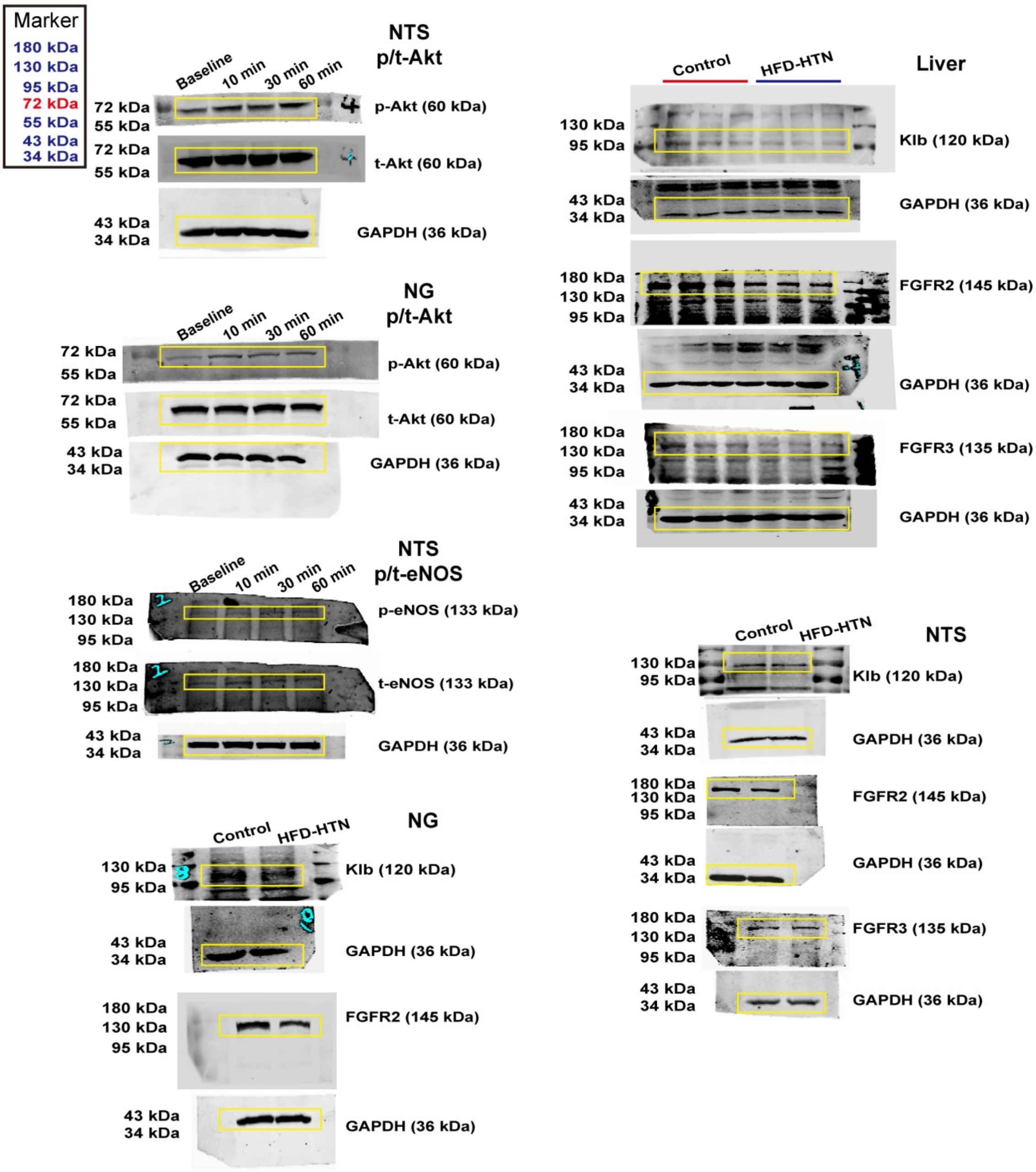

Figure S7. Full-length blots in this study were presented. The representative gels have been cropped (yellow box) for clarity; the bands were confirmed by the comparison with full-length gel images and molecular weight (marker, kDa).
